# Fronto-striato network function is reduced in major depressive disorder

**DOI:** 10.3389/fpsyt.2024.1336370

**Published:** 2024-03-06

**Authors:** Reoto Kijima, Keita Watanabe, Naomichi Okamoto, Atsuko Ikenouchi, Hirofumi Tesen, Shingo Kakeda, Reiji Yoshimura

**Affiliations:** ^1^ Department of Psychiatry, University of Occupational and Environmental Health, Fukuoka, Japan; ^2^ Department of Radiology, Kyoto Prefectural University of Medicine, Kyoto, Japan; ^3^ Department of Radiology, Graduate School of Medicine, Hirosaki University, Hirosaki, Japan

**Keywords:** major depression, reward system, striatum, prefrontal cortex, first episode

## Abstract

**Introduction:**

Major depressive disorder (MDD) is a major cause of poor quality of life and disability and is highly prevalent worldwide. Various pathological mechanisms are implicated in MDD, including the reward system. The human brain is equipped with a reward system that is involved in aspects such as motivation, pleasure, and learning. Several studies including a meta-analysis have been reported on the reward system network and MDD. However, to our knowledge, no studies have examined the relationship between the reward system network of drug-naïve, first-episode MDD patients and the detailed symptoms of MDD or age. The fronto-striato network (FSN) is closely related to the reward system network. The present study primarily aimed to elucidate this point.

**Methods:**

A total of 89 drug-naïve first-episode MDD patients and 82 healthy controls (HCs) patients were enrolled in the study. The correlation between the FSN and age and the interaction between age and illness in the FSN were investigated in 75 patients in the MDD group and 79 patients in the HC group with available information on the FSN and age. In addition, the association between the FSN and the total scores on the 17-item Hamilton Rating Scale for Depression (HAMD-17) and scores in each symptom item was analyzed in 76 MDD subjects with information on the FSN and HAMD-17. The significance of each result was evaluated according to a p-value of <0.05.

**Results:**

Age was inversely correlated with the FSN (p=2.14e-11) in the HC group but not in the MDD group (p=0.79). FSN varied with the presence of MDD and with age, particularly showing an interaction with MDD and age (p=1.04e-08). Specifically, age and the presence or absence of MDD each affected FSN, but the effect of age on FSN changed in the presence of depression. FSN did not correlate with total HAMD-17 scores or scores in each item.

**Discussion:**

The reward system may be dysfunctional in patients with MDD. In addition, the effect could be greater in younger patients. Meanwhile, there is no correlation between the function of the reward system and the severity of MDD or the severity of each symptom. Thus, the reward system network may be an important biological marker of MDD, although careful consideration should be given to age and its association with the severity of the disorder.

**Conclusion:**

The reward system function is decreased in MDD patients, and this decrease may be more pronounced in younger patients, although further research is still needed.

## 1 Introduction

Major depressive disorder (MDD), which presents with symptoms including depressed mood, decreased interest in activities, and reduced experience of pleasure, is a leading cause of reduced quality of life and disability. According to the World Health Organization, depression affects more than 300 million people worldwide, accounting for approximately 4.4% of the population (1). It is multifaceted and involves a combination of genetic, environmental, and neurobiological factors. For example, altered neurotransmission and abnormalities in the hypothalamus–pituitary–adrenal axis related to chronic stress, inflammation, reduced neural plasticity, and network dysfunction have been reported (2).

Humans and many other animals have a neural circuit called the reward system that is activated when a need is satisfied or is expected to be satisfied and produces a pleasant sensation in the individual. The reward system is involved in key components of behavior such as motivation, pleasure, and learning (3). The reward system uses dopamine as its primary neurotransmitter (4), and it consists of a network involving the ventral tegmental area of the midbrain, nucleus accumbens and posterior striatum of the basal ganglia, amygdala, and cingulate cortex of the limbic system, and frontal association areas among other areas of the frontal lobe (5, 6). The basal ganglia are involved in reward responses, behavioral choices, learning, and memory (7), while the frontal lobes are involved in reward-based decision-making, cognitive control, and emotion regulation (8).

The pathophysiology of MDD is unlikely to result from a single brain region or neurotransmitter system, and MDD is now conceptualized as a multidimensional system-level disorder affecting discrete but functionally integrated pathways (9). One important factor of this has been suggested to be a possible abnormality in the neural circuitry of MDD. Particularly, a link between MDD and the reward system has been noted. Neuroimaging studies have pointed to dysfunctions in the prefrontal cortex and striatum, which regulate the limbic system and brainstem structures involved in mediating emotional behavior, during the development of MDD (10). In addition, patients with MDD have reduced strength of functional connections between the ventral striatum and the ventral medial prefrontal and anterior cingulate cortices (11), which may be related to abnormalities in reward processing, motivation, and anhedonia. There are five cortico-basal ganglia loop circuits, namely, the motor loop, oculomotor loop, dorsolateral prefrontal loop, lateral orbitofrontal loop, and anterior cingulate gyrus loop circuits (12, 13). A recent meta-analysis demonstrated dysfunctions of reward processing behavior in MDD, demonstrating that depression was associated with small to moderate reward-processing impairments and of varying magnitudes across several reward-processing subdomains (14). This is important because the cognitive and neural mechanisms underlying reward processing and its subdomains are relatively well understood (14). Therefore, the reward system function may be a biological marker for MDD, and interventions that improve the reward system function may be effective in treating MDD. Further, the reward system may be a new therapeutic target. We previously used structural imaging to investigate the structural covariance network in the brain and extracted the fronto-striato network (FSN) (15). This network consists of the striatum and prefrontal cortex and is closely associated with the reward system (16).

Although an association between MDD and the reward system based on the fronto-striato-parietal network has been suggested, to our best knowledge, no study has investigated the effects of first-onset, drug-naive MDD and age on the fronto-striato-parietal network. Recent evidence supports that the effect of age goes beyond the prefrontal cortex and includes adaptive connectivity changes in the fronto-striato-parietal network (17). Thus, age may influence the FSN. Therefore, this study aimed to investigate the influence of MDD and age on FSN, as well as the association between FSN and the severity of MDD and each symptom, using the structural connectivity method in first-episode, medication-naïve MDD patients and healthy subjects.

## 2 Materials and methods

### 2.1 Participants

MDD patients were recruited from the university hospital of the University of Occupational and Environmental Health, Japan. Consecutive patients presenting at the Occupational and Medical University Hospital with a first episode of MDD and no medication use were recruited. MDD was diagnosed through a fully structured clinical interview using the Diagnostic and Statistical Manual of Mental Disorders, Fourth Edition, Text Revision Research Edition, and the Structured Clinical Interview for DSM Disorders Non-Patient Version. The inclusion criterion was never meeting the Diagnostic and Statistical Manual of Mental Disorders, Fourth Edition, Text Revision criteria for Axis I disorders during a psychiatrist interview. The exclusion criteria were as follows (1): mild cognitive impairment as assessed using the Mini-Mental State Examination (2); Mini-Mental State Examination scores of <27 (3); history of neurological disease or the presence of Axis I (e.g., schizophrenia, other affective disorders) or Axis II (e.g., personality disorders, mental retardation) psychiatric disorders (4); comorbid substance use disorders; and (5) unwillingness to provide informed consent. Depression severity was assessed using the 17-item Hamilton Rating Scale for Depression (HAMD-17) (18). None of the MDD patients in the study had a previous episode of mood disorder. Healthy controls (HCs) were recruited from the neighborhood; the HC group had never been diagnosed with a mental illness based on the findings of the SCID.

This study was approved by the Ethical Review Board of our institution and was conducted in accordance with the principles of the Declaration of Helsinki. Written informed consent was obtained from all patients prior to their participation in this study.

### 2.2 MRI acquisition

Magnetic resonance imaging was performed using a 3T MR system (Signa EXCITE 3T; GE Healthcare, Waukesha, WI, USA) equipped with an eight-channel brain phased-array coil. Rather than assessing functional connectivity at rest, this study utilized a structural covariance network based on brain structural imaging. The original T1 images were acquired using three-dimensional (3D) fast-spoiled gradient-recalled acquisition in a steady state. The acquisition parameters were set as follows: repetition time, 10 ms; echo time, 4.1 ms; inversion time, 700 ms; flip angle, 10°; field-of-view, 24 cm; section thickness, 1.2 mm; and resolution, 0.9 × 0.9 × 1.2 mm. All images underwent correction for image distortion due to gradient non-linearity using the Grad Warp software program (19) and for intensity inhomogeneity with the “N3” function (20).

### 2.3 Network extraction

**Figure 1:**
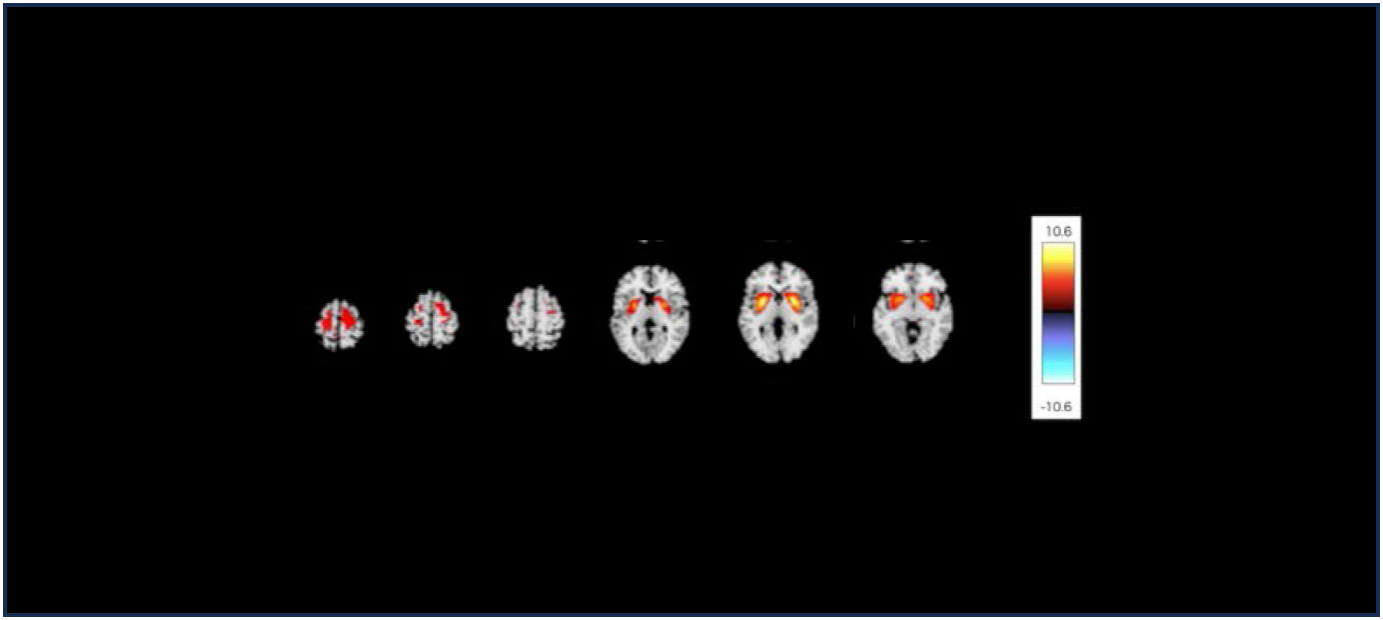
The structural covariance network of the fronto-striato network with |Z|>2.5. The red/yellow colors correspond to regions in which the voxel volumes show a positive correlation.

In this study, 3D T1-weighted images were used to analyze the structural covariance network. we initially employed a data-driven approach using the network extraction method described in our previous study. This method leverages Source-based Morphometry and Independent Component Analysis (ICA) to identify naturally occurring covariance patterns across brain regions. First, gray matter segmentation, normalization, and modulation were analyzed using Statistical Parametric Mapping 12 (Institute of Neurology, London, UK) software, employing a fully automated method as described by Ashburner (21, 22). The resulting modulated gray matter images were smoothed using an 8-mm full-width-at-half-maximum Gaussian kernel. Subsequently, the GIFT toolbox (https://icatb.sourceforge.io/groupica.htm) with minimum length was employed to estimate the independent components from all modulated gray matter images of HCs and patients with MDD. ICA was performed using a neural network algorithm (Infomax), and reliability was ensured by repeating the ICA 20 times using the ICASSO algorithm (https://research.ics.aalto.fi/ica/icasso/). The source matrix was used to determine the association between IC and voxels, whereas the mixing matrix included a loading coefficient to illustrate the relationship between each subject and each component. Sixteen networks were extracted based on the required minimum description length. The source matrix was then converted back into a 3D image to visualize structural networks, scaled to unit standard deviations (Z maps), and defined as |Z|>2.5. A neuroradiologist reached a consensus to delineate the network representing the FSN (**Figure 1**). To improve the quality of the network images, a detailed list of coordinates and regions for each network was added (**Table 1**). This clarified the specific brain regions and their coordinates for each network, including the FSN, and ensured that the reward network was accurately represented. This also clarified the distinction between ventral-frontal-striatal and dorsal-frontal-striatal areas.

**Table 1:** Structural covariance networks.

Anatomical regions	Volume (cc) left/right	Max z-value for left/right hemisphere (Talairach coordinates x, y, z)
Transverse temporal gyrus	1.0/1.0	11.9 (-42, -30, 13)/5.1 (46, –23, 11)
Superior temporal gyrus	11.5/6.7	11.5 (-43, -31, 17)/6.5 (53, -10, 3)
Insula	3.8/3.6	10.6 (-46, -31, 20)/6.1 (45, -27, 19)
Inferior parietal lobule	4.5/2.6	8.4 (-52, -35, 24)/6.2 (52, -29, 24)
Postcentral gyrus	1.7/2.6	8.3 (-52, -27, 18)/5.5 (55, -28, 21)
Sub-gyral	1.8/0.6	6.7 (-40, -34, 22)/4.9 (42, -27, 22)
Precentral gyrus	0.8/3.0	4.7 (-46, -13, 8)/4.9 (55, -7, 6)
Middle temporal gyrus	2.1/0.4	4.4 (-56, -5, -5)/4.6 (58, -2, -4)

### 2.4 Statistical analysis

Pearson’s correlation coefficient was used to examine the correlation between the FSN and age in the MDD and HC groups. In addition, in each group, FSN was used as the dependent variable and age and sex as independent variables, and multiple regression analysis was performed to check the p-value, thereby adjusting for the effect of sex in the two groups. We also analyzed the interaction of age and disease status in relation to FSN after adjusting for sex. Spearman’s correlation coefficient was used to examine the correlation between the FSN and each HAMD-17 item. To eliminate the problem of multiple comparisons, the results were processed using the Benjamini–Hochberg method. All statistical analyses were performed using EZR software version 4.0.2 (Developer: Kanda, Y.; Address: Saitama Medical Center, Jichi Medical University, Saitama, Japan), with p-values less than 0.05 considered statistically significant.

## 3 Results

A total of 89 patients with drug-naïve first-episode MDD and 82 HCs were enrolled. Overall, 75 patients in the MDD group and 79 individuals in the HC group for whom information on age and FSN was available were included in the analysis of the correlation between FSN and age and the interaction between age and disease on FSN. To analyze the association between the FSN and HAMD-17, 76 patients in the MDD group with information on the FSN and HAMD-17 were included. The primary background factors are listed in **Table 2**.

**Table 2:** Background characteristics of patients with MDD and healthy controls.

	MDD patients (n=89)	Healthy controls (n=82)	p Value
Age (years)	54.78 ± 16.23 (n=86)	35.40 ± 12.05 (n=82)	<0.01*
Males/females	39/47	56/26	0.03*
FSN	-0.24 ± 0.93 (n=77)	0.23 ± 1.02 (n=79)	0.03*
Total HAMD-17 score (0–52)	21.71 ± 7.32 (n=83)	−	

*Statistically significant (p<0.05).

MDD, major depressive disorder; FSN, fronto-striato network; HAMD-17, 17-item Hamilton Rating Scale for Depression.

### 3.1 Effects of depression and age on FSN

**Figure 2:**
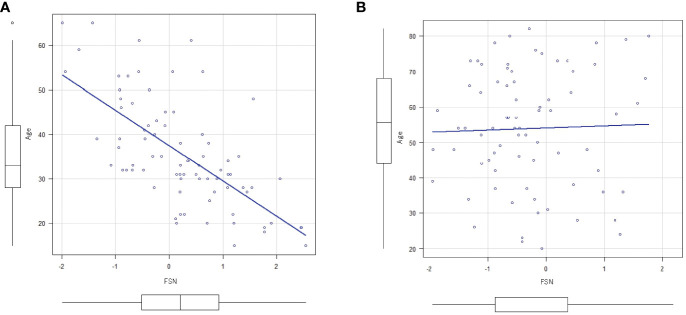
Correlation between FSN and age. **(A)** There is an inverse correlation between FSN and age (correlation coefficient=0.666, p<0.01) in the HC group. **(B)** There is no inverse correlation between FSN and age (p=0.79) in the MDD group (bottom row). FSN, fronto-striato network; HC, healthy controls; MDD, major depressive disorders.

**Figure 2** shows the results of the correlation analysis between age and FSN in the HC and MDD groups. Age was significantly correlated with FSN in the HC group but not in the MDD group. Age had a significant effect on FSN in the HC group even after adjustment for sex, while age had no significant effect on FSN in the MDD group even after such adjustment (**Table 3**). The effects of age and the presence of disease on FSN are shown in **Table 4**. There was a significant interaction between age and presence of disease.

**Table 3:** Effect of age and sex on FSN.

MDD				
	Estimate	Standard error	t Value	p Value
Age	0.002283	0.006306	-0.342	0.733
Sex	-0.157289	0.211776	-0.743	0.460
HC				
	Estimate	standard error	t value	p-value
Age	-0.56556	0.007161	-7.898	1.73e-11*
Sex	-0.186593	0.184593	-1.011	0.315

*Statistically significant (p<0.05).

MDD, major depressive disorder; FSN, fronto-striato network; HAMD-17, 17-item Hamilton Rating Scale for Depression.

**Table 4:** Effects of age, sex, and presence of depression on FSN.

	Estimate	Standard error	t Value	p Value
Age	-0.056486	0.007722	-7.315	1.47e-11*
Depression	-2.608625	0.436544	-5.976	1.62e-08*
Sex	-0.171657	0.139890	-1.227	0.222
Age: Depression	0.058825	0.009700	6.064	1.04e-08*

*Statistically significant (p<0.01).

MDD, major depressive disorder; FSN, fronto-striato network.

### 3.2 Correlation of FSN with total HAMD-17 score and each HAMD-17 item score

There was no correlation between FSN and the total HAMD-17 scores or each item score (**Table 5**).

**Table 5:** Correlation between FSN and HAMD-17 total score/each item.

	Correlation coefficient	p Value
Total	-0.00545	0.962
Depressed mood	-0.261	0.0226
Feelings of guilt	-0.0902	0.438
Suicide	-0.191	0.0981
Insomnia - early	0.115	0.322
Insomnia - middle	0.196	0.0892
Insomnia - late	0.11	0.345
Work and activities	-0.28	0.0144
Retardation - psychomotor		
Agitation	0.0847	0.467
Anxiety - psychological	-0.117	0.314
Anxiety - somatic	0.0317	0.786
Somatic symptoms GI	-0.173	0.135
Somatic symptoms - General	0.146	0.208
Sexual dysfunction - menstrual disturbance	0.115	0.323
Hypochondria	0.33	0.00364
Weight loss by history	0.00895	0.939
Insight	0.184	0.111

p-Value adjusted by Bonferroni method=0.00278.

HAMD-17, 17-item Hamilton Rating Scale for Depression.

## 4 Discussion

This study compared the relationship of FSN with age between healthy subjects and patients with depression and analyzed the effects of the presence of MDD, age, and their interactions on FSN. The results showed an inverse relationship between FSN and age in HCs, whereas this relationship did not exist in patients with MDD. Furthermore, the FSN was significantly affected by both the presence of disease and age, indicating a significant interaction between the two.

The inverse relationship between FSN and age in HCs suggests that the function of this network diminishes with age. A previous study, in which network extraction was performed by functional magnetic resonance imaging (fMRI), also found a negative association between age and brain network connectivity, including the default mode network that contains the superior and middle frontal gyri, posterior cingulate, middle temporal gyrus, and superior parietal region (23). Meanwhile, the FSN is involved in reward processing, motivation, and decision-making in patients with MDD. However, this inverse correlation was not found in patients with MDD in the current study. This indicates that the younger the patient with MDD, the lower the FSN function and the lower the age-related correlation. Considering the inverse correlation found in HCs, these results suggest that in MDD, the FSN function is lower in younger patients and this phenomenon is no longer present as aging progresses. This indicates that MDD has a neurobiological basis and that abnormalities in the FSN, which are assumed to be related to the reward system, contribute to depressive symptoms. Further, FSN dysfunction may be a biological marker for the diagnosis for MDD. A large amount of evidence indicates a link between MDD and the reward system. Blood flow differences have been observed in regions associated with the dopaminergic system (24), and the levels of homovanillic acid, a dopamine metabolite, are decreased in the cerebrospinal fluid and transvenous plasma of patients with MDD (25, 26). The noted changes in central dopaminergic function in MDD provide indirect evidence of dysfunction of the reward system in MDD.

fMRI studies have demonstrated that dopaminergic neurons project from the ventral tegmental area of the midbrain to several brain regions, including the nucleus accumbens (25, 26). One fMRI study showed that MDD patients on long-term medication have reduced responses to reward learning signals, particularly in the ventral striatum and anterior cingulate gyrus (27). Functional neuroimaging studies of patients with MDD have shown that ventral striatal regions, such as the nucleus accumbens, are less active, and orbitofrontal cortex activity is elevated during reward tasks (28). The reward system is a network of multiple regions, and reports indicate that all regions comprising the reward system are altered in patients with MDD, providing indirect evidence for reward system dysfunction in MDD. The reward system is considered a network in the brain, and some studies have indicated to a link between MDD and the reward system network. A resting-state fMRI study focused on the nucleus accumbens-based reward system circuitry in patients with MDD confirmed the important role of reduced functional coupling in the reward network in the neuropathology of MDD (29). Our report focused on how age affected the association between MDD and the reward network, and the findings may help in further understanding the relationship between MDD and reward system dysfunction. The interaction between age and the presence of disease shown in this study indicated that MDD may have a specific effect on age-dependent changes in the FSN. This underscores the importance of considering age in the treatment and management of MDD and suggests the possible need for an individualized approach for patients with depression in different age groups.

The current study also found that the FSN was not correlated with the total HAMD-17 score or each item score in MDD patients. The ability to predict when and where rewards will occur plays an important role in human positive behavior. Neuroimaging studies suggest that the amygdala, orbitofrontal cortex, and ventral striatum are involved in reward prediction (30, 31). To select a different behavior from multiple behavioral options, the predicted rewards associated with each behavior must be compared and evaluated, and the behavior with the highest reward among the predicted rewards must be selected. Involvement of the orbitofrontal cortex has also been suggested for this selection (7). Given the involvement of the reward system in motivating behavioral choices, it appears that dysfunction of the reward system may make it difficult for subjects to motivate their behavior. In addition, the ventral tegmental area of the midbrain projects dopamine neurons to the striatum and prefrontal cortex, as well as to the amygdala and hippocampus, which are involved in emotion (32). From this perspective, FSN may be associated with depressive mood, a core symptom of depression. Loss of pleasure is a major symptom of MDD (33). However, a recent systematic review of fMRI-based studies indicates that impairment of the reward system, as indicated by hypoactivation of the striatum and blunted frontal lobe sensitivity, is associated with impaired reward processing in MDD (34). This suggests that impairment of the reward system is associated with depressive mood and loss of pleasure, which are core symptoms of depression.

However, the present study found no correlation between FSN and total HAMD-17 scores or individual item scores in MDD patients. MDD is a highly heterogeneous syndrome based on a complex pathology with a wide variety of phenotypes. Particularly, although the FSN plays an important role in the pathophysiology and symptoms of depression, it may also work in complex associations (or collaborations) with other neural networks to create various phenotypes of depression. Our previous study found no significant differences in the salience, medial temporal lobe, default mode, medial temporal lobe, default mode, and central executive network between the MDD and HC groups (15). This could be one of the reasons for the lack of association with the total HAMD-17 score and each item score in the current study. The present results may reflect that the pathophysiology of depression is due to dysfunction of multiple brain networks and not only of the FSN (35). The association of brain structure and function with complex behaviors should be investigated in large-scale studies to ensure reliability (36). Considering the previous studies on the association between reward system network impairment and depressive symptoms, it is possible that the current study did not have an adequate sample size, which may have affected the results.

### 4.1 Limitations

This study had several limitations. As this was a cross-sectional study that enrolled a small number of patients, the temporal relationship between depressive symptoms and the frontal-basal ganglia network remains unknown. Some of the MDD subjects in this study received psychotherapeutic treatments such as cognitive-behavioral therapy and dynamic psychotherapy, and these treatments may have influenced the results. Considering that the relationship between brain structure and function and complex behavior should be investigated using large-scale studies to ensure reliability (36), the sample size was not sufficient in the current study. In addition, the FNS was determined through subjective visual assessment in the study subjects in whom the brain volumes were highly interlinked. Although the FSN is classified into five loop circuits (12, 13), it is unclear which circuit the present network falls into. Although the results indicated a significant difference in age between the HC and MDD groups, the analysis of the correlation between FSN and age in each group did not consider the imbalance in age between the two groups. This may have affected the conclusions. The current study did not obtain information on the duration of MDD symptoms. Although the disease duration would not be long because the patients only had their first episode and were untreated, the duration may still have influenced the results. In addition, although the frontal-basal ganglia network includes the nucleus accumbens and the prefrontal cortex, which are parts of the reward system, it may also include other areas that are not related to the reward system. Therefore, the representativeness of the frontal lobe-basal ganglia network as an evaluative value of the reward system has not been fully elucidated. In addition, the correlations were weak, and the present study intended to demonstrate correlations, not causality. Further studies are required to ascertain whether these correlations are clinically meaningful or therapeutically useful.

### 4.2 Conclusion

The function of the reward system is decreased in patients with MDD, and the extent of this decrease may be more pronounced in younger patients. Meanwhile, the overall severity of MDD and each severity are not related to the decline in reward system function. However, age may need to be taken into consideration. In addition, the usefulness of using the severity of the disease may need to be carefully judged. Further studies are needed to validate these findings.

## Data availability statement

The raw data supporting the conclusions of this article will be made available by the authors, without undue reservation.

## Ethics statement

This study was conducted in accordance with the Declaration of Helsinki, and the protocol was approved by the Ethics Committee of the University of Occupational and Environmental Health, Japan, Kitakyushu, Japan (approval number: H25-13). Informed consent was obtained from all subjects involved in the study.

## Author contributions

RK: Conceptualization, Data curation, Formal Analysis, Investigation, Project administration, Software, Validation, Visualization, Writing – original draft, Writing – review & editing. KW: Writing – original draft, Writing – Review & editing. NO: Methodology, Software, Validation, Writing – review & editing. AI: Investigation, Software, Supervision, Writing – review & editing. HT: Investigation, Methodology, Software, Writing – review & editing. SK: Methodology, Software, Supervision, Writing – review & editing. RY: Conceptualization, Funding acquisition, Project administration, Supervision, Writing – review & editing.
